# Sequential targeting of PI3Kδ and LAG3 as an effective anti-cancer approach

**DOI:** 10.1038/s41416-021-01285-1

**Published:** 2021-04-06

**Authors:** Sarah N. Lauder, Bart Vanhaesebroeck, Awen Gallimore

**Affiliations:** 1 Division of Infection and Immunity, Cardiff University School of Medicine, SIURI, Cardiff, UK; 2 UCL Cancer Institute, Paul O’Gorman Building, University College London, London, UK

**Keywords:** Tumour immunology, Immunotherapy, Tumour immunology

## Abstract

Emerging studies have demonstrated the potential of PI3Kδ blockade as an immunotherapy for solid tumours. In pre-clinical models, we recently demonstrated that anti-LAG3 immune checkpoint blockade vastly potentiated PI3Kδ-based immunotherapy, enabling successful tumour control in all treated mice.

## Main

Immunotherapies that unleash powerful anti-tumour T cell responses, such as anti-PD1/PD-L1 or anti-CTLA4 therapy can have impressive clinical benefit, albeit only in a small proportion of patients. These therapies aim to improve the potency of the anti-tumour response and/or to increase the frequency of tumour-infiltrating CD4^+^ and CD8^+^ T cells, often by restoring the balance of immunosuppressive Tregs to anti-tumoural CD4^+^ and CD8^+^ T cells.^1^ While studies designed to either inhibit or deplete regulatory Tregs have shown promise at the pre-clinical stage, developing clinically-relevant Treg-specific therapies have proved more challenging. Targeting Tregs by blocking the leukocyte-enriched PI3Kδ is an attractive target for therapy given that, compared with conventional T cells, Tregs are substantially more dependent on PI3Kδ activity, with PI3Kδ inhibition dampening their proliferation and suppressive functions.^2–6^

Using genetically engineered mice with systemic or Treg-selective PI3Kδ inactivation, Ali and colleagues previously demonstrated increased resistance to tumour growth, while pharmacological inactivation using the PI-3065 PI3Kδ inhibitor provided partial tumour control in treated animals.^6^ Using this PI3Kδ inhibitor, we demonstrated that while all treated animals exhibited some level of tumour control, these mice fell into two groups namely those that showed only slowed tumour growth, which we refer to as non-regressors (PI-3065 NR), and those that showed significantly reduced tumour burden (regressors; PI-3065 R).^7^ Investigating the differences between these two groups, we found that complete control of tumour burden was reliant on: (i) the dampening of a Treg response and (ii) the generation of an improved antigen-specific CD8 response.

**Fig. 1:**
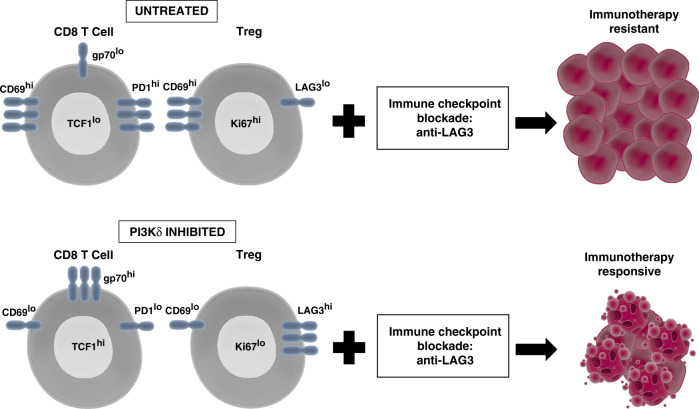
Schematic showing changes to the tumoural T cell infiltrate and response to immunotherapy following PI3Kδ blockade. Pharmacological inhibition of PI3Kδ results in a significant enrichment of antigen-specific CD8 T cells with improved metabolic fitness and self-renewal capacity and a significant reduction in the number of tumoural Tregs, thereby increasing the ratio of CD8:Tregs within the tumour. The remaining Tregs express high levels of LAG3, which renders tumours responsive to additional immune checkpoint blockade with anti-LAG3 antibodies.

Intra-tumoural effector T cells were re-invigorated following PI3Kδ inhibition as demonstrated by improved metabolic fitness and enhanced self-renewal capacity. The significant reduction in tumour burden observed in PI-3065 R mice was driven by a marked expansion of tumour antigen-specific CD8 T cells and resistance to exhaustion as evidenced by decreased PD1 expression^7^ (Fig. 1).

Both in PI-3065 NR and R mice, the number and proportion of Tregs within the tumour-infiltrating lymphocyte pool were significantly reduced compared with vehicle-treated mice. However, it was differences in the Treg phenotype that delineated which mice displayed tumour regression. Indeed, compared with PI-3065 R mice, PI-3065 NR tumours contained a pool of highly-proliferative Tregs, identified by high expression of Ki67, CD69 and LAG3.^7^

The role of LAG3 in immune regulation, including the generation of effective anti-tumour immune responses remains poorly understood. LAG3 is expressed on activated CD4^+^ and CD8^+^ T cells and Tregs, and its surface expression is associated with downmodulation of T cell responses.^8^ While LAG3 expression on Tregs is necessary for their suppressive function^9^ and an infiltration of LAG3^+^ T cells is associated with poor prognosis for a range of human tumours, studies to date have shown that LAG3-blockade or genetic ablation in mice confers negligible effects on tumour growth.^10^ However, anti-LAG3 therapy in combination with a second immune checkpoint blockade, particularly anti-PD1, can significantly reduce tumour growth. These studies suggest that blockade of LAG3 alone is not sufficient to counteract the immunosuppressive tumour micro-environment. To determine if the increased pool of LAG3^+^ Tregs observed in PI-3065 NR mice were enabling suppression within the tumour micro-environment and preventing full tumour control, mice were given anti-LAG3 antibody therapy once tumours were palpable, in combination with PI3Kδ blockade. This combination therapy resulted in significantly reduced tumour burden, with half of the treated animals eradicating their tumours (Fig. 1). To further probe the therapeutic potential of the combination of anti-LAG3 and PI3Kδ inhibition, we commenced both treatments in established tumours and found that combination therapy was able to reduce tumour burden, with some animals able to eradicate established tumours. We found that increased LAG3 expression was restricted to Tregs within the tumour and was not observed in the draining lymph nodes or spleen, indicating that anti-LAG3 antibody therapy acts directly within the tumour.

However, not all tumour models tested responded to PI3Kδ inhibition, highlighting the importance of examining the T cell response to identify tumour-specific impediments to therapy. We found that PI3Kδ-responsive tumours were characterised by an increased CD8:Treg ratio upon PI3Kδ inhibition, and this turned out to be an essential requisite to tumour control. In the absence of such an increased ratio, tumours remained resistant to subsequent therapy with anti-LAG3. In other words, some degree of primary anti-cancer immune response is required in order to achieve an anti-tumour effect of PI3Kδ and LAG3 combination therapy.

Several PI3Kδ inhibitors are now progressing in clinical trials to determine their potential utility as an immunotherapy for solid malignancies. Recent data have also rekindled the early observation that, in addition to its high constitutive expression in leukocytes, PI3Kδ can also become highly expressed in some solid tumour cell types, such as breast and melanoma.^11^ While the functional role of this cancer-cell-intrinsic PI3Kδ is not well-established, recent evidence has been presented that PI3Kδ inhibition can also have direct anti-tumour effects in additional to its immunostimulatory effects (reviewed in ref. ^12^).

PI3Kδ inhibitors in clinical trials include AMG-319 as a monotherapy in a window-trial in head and neck cancer (Amgen and Cancer Research UK; NCT02540928; completed—results to be reported) or in combination with anti-PD1 immune checkpoint therapy in a range of advanced solid tumour indications (parsaclisib from Incyte; NCT02646748/NCT03589651), with iOnctura testing their PI3Kδ inhibitor IOA-244 as monotherapy and in combination with the chemotherapeutics pemetrexed/cisplatin in a range of advanced solid tumour indications (NCT04328844). Elucidating the effect of PI3Kδ inhibition on the immune response within each tumour type, specifically the anti-tumour CD8 and Treg response, will be key in determining which tumours are likely to be responsive to PI3Kδ inhibition. In those tumours that are responsive, detailed phenotyping of the tumour-infiltrating lymphocytes has the potential to highlight which co-inhibitory receptors should be targeted with additional immune checkpoint blockade to improve the clinical response.

## Data Availability

All data within this article are available through the cited references.
